# Case of Intus-Susception, Successfully Treated

**Published:** 1835-07-01

**Authors:** John Howship

**Affiliations:** Lecturer on Surgery in the Medical School at the Charing Cross Hospital, and Surgeon to that Hospital.


*To the Editor of the Medical Quarterly Review.*

Sir: Should the following case be deemed sufficiently valuable to entitle it to publication, I shall be happy to see it recorded in your ably conducted Review.

I am, sir, your obedient servant,

J. Howship.

*Saville Row, June* 15, 1835.

*Case of Intus-susception, successfully treated.* By John Howship, Lecturer on Surgery in the Medical School at the Charing Cross Hospital, and Surgeon to that Hospital.

In the delivery of my lectures on surgery I have, for some years, after describing the symptoms and treatment of strangulated hernia, been in the habit of pointing out the modes of discriminating those cases in which the symptoms of hernia are present, without the tumour; cases of internal stricture, or strangulation; concluding my observations on these obscure and complicated diseases by some notice of intus-susception, an affection which, when it has induced symptoms, almost invariably destroys life. On these occasions I have, among other cases, referred to one ending fatally, published by me in the Edinburgh Medical and Surgical Journal, shewing the preparation illustrating that case, and lastly have mentioned the following case, as one in which the symptoms were such as clearly identified the disease in the judgment of the practitioner first consulted, as well as in my own opinion; a case in which mechanical distention of the lower part of the intestinal tube succeeded in reaching the affection, removing the complaint, and consequently saving the life of the patient.

Tuesday, May 13, 1828. I was requested to see Miss J. W., a little girl about four years old, considered to be very seriously ill, having been seized on the preceding evening with pain in the belly, sickness at stomach, frequent and severe straining to go to stool, yet passing only blood, per anum. On seeing the child I found the above symptoms still existed, with quick and hard pulse, hot skin, white tongue, tension and tenderness of the abdomen. Finding she was already under the care of Mr. Plumbe, who had directed the necessary treatment, I recommended its continuance till the following day, to have the opportunity of meeting that gentleman in consultation.

Wednesday, May 14. I met Mr. Plumbe, who stated that he had already applied leeches, tried an enema, which had immediately returned, and ordered aperient medicine, which was rejected; adding that from the symptoms, and especially the obstinate costiveness, he thought there was strong reason to suspect the existence of intus-susception; in which opinion I fully coincided. Aperient medicines and a warm bath were ordered.

Thursday, 15th. There was still no passage through the bowels, and all the medicine had been rejected as soon as taken. The pulse was now 160; the abdomen rather less tense and tender than on the preceding day. We both agreed as to the certainty (as far as it could be proved) of the existence of intus-susception of some part of the intestinal tube, and also as to the absolute certainty of the child's death, unless something more was attempted for her relief.

In this dilemma, I suggested that the introduction of a volume of warm water might possibly prove useful, provided the intus-susception was situated, as it generally is, in the great intestine; and that even if the affection was beyond the reach of this means, it could scarcely operate prejudicially, in a case obviously passing on to a fatal termination. This suggestion having been considered and approved, we met at seven, P.M., to carry it into effect.

The fluid injected was warm gruel. I used Weiss's syringe, introducing the fluid very slowly, in order gradually to relax the intestine; the sides of the sphincter being pressed upon and supported, to prevent the return of the fluid, without which assistance the operation would have failed altogether.

At intervals the progress of the operation was suspended, to allow the pains from distention to subside; the purpose of the operation being also occasionally assisted by gentle frictions about the abdomen; and then more fluid was introduced, until we estimated the injected volume at between two and three pints. In fifteen minutes after the conclusion of the operation (which had occupied an hour), the child was allowed gradually to relieve the bowels, and in the liquid that flowed several soft fæcal masses were found. While the load of fluid was retained, retching came on, but nothing was thrown off from the stomach; the pulse fell to 110, but soon afterwards quickened again.

A senna mixture, in small doses, was directed to be given at short intervals through the night.

Friday, 16th, two P.M. The pains in the belly, which with much severity had recurred every half-hour during the night previous to the operation, had only returned since that time once or twice, and then only very slightly. The calls to make water, also, were much less frequent than before; there was less tenderness of abdomen, and the child was more lively. The pulse was 130, and the abdomen still full, though soft.

The medicines, from the time of the operation, had uniformly remained on the stomach, and three hours after the administration of the injection, a fluid, bilious and fæcal motion, nearly a pint in quantity, had passed. The child was considered better, and the purging mixture directed to be continued, and a saline mixture to be also given.

Saturday, 17th. No return of pain, slight tenesmus, and two small watery motions. It was supposed that the bowels were now quite clear, but the aperient medicine was continued.

Sunday, 18th. The medicine three times taken, nearly two pints of solid pulpy fæces had passed the bowels, much to the surprise of the parents, who said it must have remained from the commencement of the attack, as the child had taken absolutely nothing since. The pulse was still 130, but she had slept well, and was quite easy.

Monday, 19th. She was active, hungry, and running about in excellent spirits, and, in short, entirely recovered; and has from that time to the present date enjoyed uninterrupted good health.

It is scarcely necessary to add, that the principle on which mechanical distention was had recourse to in this case, was one by which it was presumed that the outer ring of the constricting intestine being of necessity induced to yield into a state of progressive relaxation, the warmth of the contained fluid would favour the relaxation, while its absolute pressure would by degrees wear out resistance; and that in this way, as the bowel relaxed itself, the whole of the included portion of the intestine might be eventually restored to its previous state of natural independence; and the event justified the assumption.

I am perfectly aware that those who are disposed to object, may easily find objections to the course adopted in the present case. The bowel might have been so far advanced in inflammation as to have prevented the success of the means used; no doubt it might, but this could have been determined only by the experiment, which would even then have left the case as it found it. Again, had the obstruction been situated in the small intestine, the valve of the colon would have rendered the attempt unavailing; this, also, is evident enough, but the trial proved that the difficulty was within reach, instead of beyond it.

A more formidable objection however, in appearance, may be started, in the chance that the bowel might have been ulcerated, and that then the pressure of the contained fluid might have induced its escape into the cavity of the abdomen, and thus have destroyed life; but independent of the infrequency of this disease, at any age, its existence is less to be suspected in children, and least of all in a child obviously exhibiting every possible proof of good health, up to the very hour of the sudden attack, and it therefore would appear hardly reasonable to consign a patient over to certain death, because the means proposed, that may probably rescue him from destruction, may, by a bare possibility, in one such case out of ten thousand, prove a source of fresh danger.

